# Concentration-Response Relationship between PM_2.5_ and Daily Respiratory Deaths in China: A Systematic Review and Metaregression Analysis of Time-Series Studies

**DOI:** 10.1155/2017/5806185

**Published:** 2017-10-16

**Authors:** Mengying Ren, Xin Fang, Mei Li, Sun Sun, Lu Pei, Qun Xu, Xiaofei Ye, Yang Cao

**Affiliations:** ^1^Department of Public Health Sciences, Karolinska Institutet, 171 77 Stockholm, Sweden; ^2^Unit of Biostatistics, Institute of Environmental Medicine, Karolinska Institutet, 171 77 Stockholm, Sweden; ^3^Department of Cardiology, Shanghai Changzheng Hospital, Shanghai 200003, China; ^4^Health Outcomes and Economic Evaluation Research Group, Department of Learning, Information, Management and Ethics, Karolinska Institutet, 171 77 Stockholm, Sweden; ^5^Division of Epidemiology and Global Health, Department of Public Health and Clinical Medicine, Umeå University, 901 87 Umeå, Sweden; ^6^Department of Epidemiology and Biostatistics, Institute of Basic Medical Sciences, Chinese Academy of Medical Sciences, School of Basic Medicine, Peking Union Medical College, Beijing 100005, China; ^7^Department of Health Statistics, Second Military Medical University, Shanghai, China; ^8^Clinical Epidemiology and Biostatistics, School of Medical Sciences, Örebro University, 701 82 Örebro, Sweden

## Abstract

The association between the particulate matters with aerodynamic diameter ≤ 2.5 *μ*m (PM_2.5_) and daily respiratory deaths, particularly the concentration-response pattern, has not been fully examined and established in China. We conducted a systematic review of time-series studies to compile information on the associations between PM_2.5_ concentration and respiratory deaths and used metaregression to assess the concentration-response relationship. Out of 1,957 studies screened, eleven articles in English and two articles in Chinese met the eligibility criteria. For single-day lags, per 10 *μ*g/m^3^ increase in PM_2.5_ concentration was associated with 0.30 [95% confidence interval (CI): 0.10, 0.50] percent increase in daily respiratory deaths; for multiday lags, the corresponding increase in respiratory deaths was 0.69 (95% CI: 0.55, 0.83) percent. Difference in the effects was observed between the northern cities and the south cities in China. No statistically significant concentration-response relationship between PM_2.5_ concentrations and their effects was found. With increasingly wider location coverage for PM_2.5_ data, it is crucial to further investigate the concentration-response pattern of PM_2.5_ effects on respiratory and other cause-specific mortality for the refinement and adaptation of global and national air quality guidelines and targets.

## 1. Introduction

Ambient air pollution (AAP) has become a major environmental and public health risk for human society globally. The World Health Organization's (WHO) Global Urban Ambient Air Pollution (AAP) Database indicated that 98% of cities in Low- and Middle-Income Countries (LMICs) are exposed to air pollution that far exceeds the WHO Air Quality Guidelines (AQGs) limits [1, 2]. From the Global Burden of Disease (GBD) study, air pollution was ranked as the fourth leading risk factor accounting for more than 5.5 million premature deaths across the world each year and more than 50% of all AAP-attributable deaths occurred in China and India [2, 3]. Ambient particulate matter (PM) pollution is specifically connected to 2.9 million deaths and 69.7 million disability-adjusted life-years (DALYs) in 2013 [2, 3].

Fine PM or PM_2.5_, namely, particles with aerodynamic diameter of 2.5 micrometres or less, can lodge deeply into lung passageways and enter major organ systems [4, 5]. Of all AAP, PM_2.5_ poses the greatest health risks that are closely associated with a wide variety of acute and chronic illnesses and premature deaths, but predominantly from cardiovascular and respiratory outcomes [4, 6–9]. WHO set the AQGs of an annual mean of 10 *μ*g/m^3^ and a 24-hour mean of 25 *μ*g/m^3^ for PM_2.5_, representing the upper end of PM_2.5_ concentration range below which short-term and long-term mortality risks are expected to be significantly reduced [10]. The global estimates of annual average PM_2.5_ in 2013 showed that 87% of the world's population was exposed to PM_2.5_ higher than 10 *μ*g/m^3^, with consistent increases between 1990 and 2013 in population-weighted mean concentrations particularly in Asia [11].

The geographic extent of PM_2.5_ pollution in China is unprecedented, as only 0.4% of the Chinese population lives in areas that meet the WHO AQGs [1, 11]. In 2013 alone, around 910,000 people in China died prematurely due to AAP [3] and 760,000 deaths were associated directly with PM_2.5_ [12]. Real-time air quality data using PM_2.5_ gauge in the 74 leading cities in China became officially available to public since 2012 and ambient PM_2.5_ concentration is being monitored against the National Ambient Air Quality Standards [13–16].

The health effects of AAP especially on total mortality and cardiovascular diseases have been extensively studied across different countries and regions. Given that PM_2.5_ also causes asthma, respiratory inflammation, jeopardizes lung functions, and even promotes cancers, its impact on human respiratory system should not be dismissed. However, epidemiological evidence for the PM_2.5_ pollution on respiratory mortality has not been well synthesized, especially at high concentration areas such as in China. Respiratory deaths associated with PM_2.5_ at relatively high concentrations (e.g., 24-hour mean exceeding 75 *μ*g/m^3^) were sporadically studied and previous findings presented little evidence of damage threshold of concentration range [7, 17–19]. Most previous studies either were based on weighed exposure-response coefficients from epidemiological findings in Europe and North America or remain unexamined with a focus on short-term effects based on projected or recently observed PM_2.5_ level [15, 20]. Plenty of research findings from countries with relatively low PM_2.5_ concentration range have shown linear association between PM_2.5_ exposure and premature deaths from respiratory outcomes [6–8, 21], while some studies indicated lower relative risk (RR) with high PM_2.5_ concentrations and the exposure-response curve turning flat at extremely high PM_2.5_ levels [18, 19, 22, 23]. However, these findings could not be readily applied to the AAP situations in LMICs because of the significant variations in meteorological conditions, PM sources and components, and population sensitivity to the PM_2.5_ [17–19].

As systematic PM_2.5_ data measurement in China became available and frequently used for research since 2012, recent studies looked into health effects of high PM_2.5_ concentrations by cause-specific mortality indications [18, 24–27]. A most recent publication of a nationwide time-series study in China evaluated the short-term associations between PM_2.5_ and daily mortality across 272 representative Chinese cities between January 2013 and December 2015. Comparing with similar multisite studies in Europe and North America, the findings from the study in China suggested weaker effects on daily mortality for each 10 *μ*g/m^3^ increase in PM_2.5_ concentrations [28]. A wider range of risk heterogeneity among different PM_2.5_ sources and possible confounders including meteorological variables and exposure lag-day effects has been considered [29]. It is worthwhile to systematically review the recent studies and synthesize the epidemiological evidence on the health effects of PM_2.5_ at a notably high concentration level that exceeds both WHO AQGs and interim targets. Research on PM_2.5_ data specifically in China would be an important milestone adding value to future studies in populous LMICs in addition to the findings on the relatively lower PM_2.5_ levels from Europe and North America.

Concentration-response functions drawn by meta-analysis are well accredited in supporting epidemiological evidence of the integrated information for health impact assessments [29]. A comprehensive overview of existing literature and the quantitative estimates of the correlations between PM_2.5_ and respiratory deaths in China would provide valuable feedback to the current WHO AQGs Standards over mortality effects and potentially shed light on public health strategies in other developing contexts where AAP poses major health and development threats. With the increasing availability of epidemiological studies on respiratory health effects of PM_2.5_ in China, we conducted a systematic review and meta-analysis of population-based studies in China to (1) compile and compare information from major literature databases on the fatal respiratory outcomes by PM_2.5_; (2) retrieve evidence from identified studies regarding the association of PM_2.5_ with respiratory deaths in China; (3) assess concentration-response relationships between PM_2.5_ concentrations and their health effects.

The protocol for this study was registered in the PROSPERO international prospective register of systematic reviews in September 2016 (https://www.crd.york.ac.uk/PROSPERO/; CRD42016047456). The Preferred Reporting Items for Systematic Reviews and Meta-Analyses (PRISMA) Statement and the Meta-Analysis of Observational Studies in Epidemiology (MOOSE) Statement were referred to as a basis for methodological guidance of this research [30].

## 2. Materials and Methods

### 2.1. Literature Search

The study followed PRISMA and MOOSE as search and screening guidelines. The literature review articles were retrieved from Ovid Medline (http://ovidsp.tx.ovid.com), Embase (http://www.embase.com), Web of Science Core Collection (http://webofscience.com), Ovid Global Health (http://ovidsp.ovid.com/ovidweb.cgi?T=JS&NEWS=n&CSC=Y&PAGE=main&D=cagf), and China National Knowledge Infrastructure (http://www.cnki.net) from their inception to 16th September, 2016. Karolinska University Library Service and Peking Union Medical College supported the literature search and the retrieval of English and Chinese articles, respectively. We also accessed the System for Information on Grey Literature in Europe (http://www.opengrey.eu) and Grey Literature Report (www.greylit.org) to identify potential unpublished studies. These searches were supplemented by hand searching from the references of relevant research articles.

The combinations of the following key terms for the literature search include (1) particulate matter, ambient particulate, PM_2.5_, ultrafine particulate, ultrafine particle, air pollution, air pollutants, and inhalation exposure; (2) respiratory tract diseases; (3) China, Chinese, names of major Chinese cities; (4) mortality and death. There were no language restrictions. The detailed log of search strategies with a complete list of key words and medical subject heading (MeSH) terms is shown in Boxes 1–5.

### 2.2. Inclusion Criteria and Identification of Articles

Initially, we planned to include all the population-based prospective studies and time-series studies in our systematic review; thus articles eligible for meta-analysis met the following criteria: (1) they are original, population-based studies including prospective study, cohort study, nested case-control study, time-series study, and longitudinal study; (2) the main pollutants were ambient PM_2.5_ or fine PM; (3) PM_2.5_ concentration data in China were reported; (4) the endpoint of interest was mortality/deaths from respiratory outcomes; (5) the risk estimates and the associated 95% confidence intervals (CIs) were reported; (6) the risks were adjusted for potential confounders such as geographic regions, meteorological factors, or exposure lag in days.

Studies were excluded if they (1) were reviews, editorials, commentaries, letters, methodological papers, experimental, retrospective, or cross-sectional studies; (2) exclusively focused on high-risk groups such as smokers or patients with preexisting respiratory symptoms; (3) focused on nonrespiratory deaths and exposure to household or indoor air pollution, second-hand smoke, PM_10_, gaseous pollutants including carbon monoxide (CO), ozone (O_3_), nitrogen dioxide (NO_2_), and sulfur dioxide (SO_2_).

Studies were selected for inclusion through a two-stage process. Literature search results (titles and abstract) identified by the search strategy were screened independently by two reviewers (M. R., Y. C.) to identify all citations that potentially met the inclusion/exclusion criteria detailed above. Full manuscripts of selected citations that appeared potentially relevant were obtained. These were assessed by two reviewers (M. R. and M. L.) against the inclusion/exclusion criteria using a flow chart and checked independently by the third reviewer (X. Y.) before a final decision regarding inclusion was agreed. At each stage any disagreements were resolved by discussion, with the involvement of an extra reviewer (Y. C.) when necessary.

**Figure 1:**
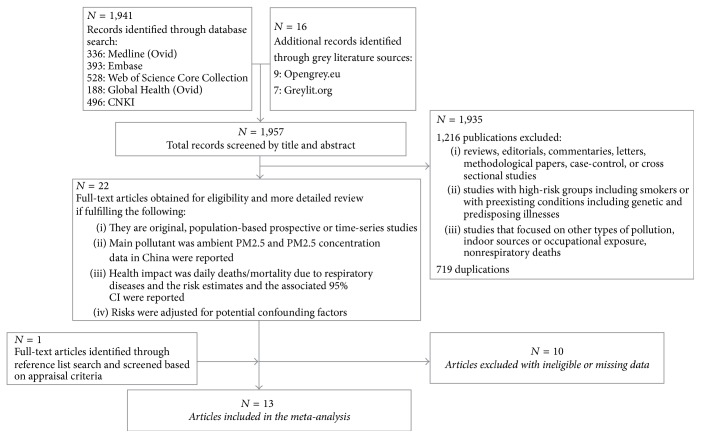
PRISMA Flowchart of literature search and screening.

Full-text articles were downloaded for all the abstracts that met the search and screening criteria and identified through inclusion criteria for further analysis. Specific literature identification steps with selection results were summarized in Figure 1.

### 2.3. Data Extraction

For the selected studies, the information on the title, authors, year of publication, study location, geographic region, duration, PM_2.5_ concentration, daily respiratory mortality, risk measurement, lag days, and adjustments was extracted and entered into a Microsoft Excel form. Two investigators (M. R. and X. Y.) independently conducted and checked the data extraction. Discrepancies in the extracted data were resolved by discussion, with involvement of the third reviewer (Y. C.) when necessary.

For PM_2.5_ concentration (*μ*g/m^3^) data, the daily average was represented by median value and supplemented by the midpoint of lower and upper boundaries or daily mean when median value was not provided. For risk measurement, percent increase in respiratory mortality per respective unit increase in daily PM_2.5_ concentration with 95% CI and standard error (SE) was extracted or calculated from relative risk (RR); that is, percent increase in respiratory mortality = (RR − 1) × 100%. Risk measurement with the regional divisions (north versus south) was recorded. Because pollution levels are often highly correlated and selecting a single best fitting lag might result in inconsistence across studies, it is important to consider the pattern of lag periods across the studies [42]. Therefore, we conducted subgroup analysis by categorizing the studies into single-day lags and multiday lags. Single-day lag means the mortality after 0, 1, or more days with exposure to the PM_2.5_ concentration of the exposure day. Multiday lag means the mortality after 1 or more days with exposure to the moving average PM_2.5_ concentration of 2 or more days. For studies having several single-day or multiday lag effects, the average effects were used in the synthesis. If the risk effects for a study were estimated in more than one model, only the overall value with adjustment for fewer confounders was used for pooled analysis to allow for higher homogeneity among the included studies.

### 2.4. Statistical Analysis

For consistency and uniformity of comparison, the percent increase in respiratory mortality per 10 *μ*g/m^3^ increase in PM_2.5_ concentration was used for risk estimates in the pooled analysis. Studies providing RR or per IQR increase in PM_2.5_ concentration were converted into the aforementioned equivalent risk estimates.

The statistic *I*^2^, a quantitative measure of inconsistency, was calculated to evaluate the statistical heterogeneity across studies [43]. *I*^2^ > 30% is considered moderate heterogeneity and *I*^2^ > 50% is considered substantial heterogeneity [44]. Both fixed- and random-effects meta-analysis were used when heterogeneity occurs across studies. Potential publication bias was assessed by Egger's test. Subgroup analysis for different lag-day structure (single-day lags and multiday lags) was conducted. Division by geographic regions (northern cities versus south cities) for the lag structures was tested for possible additional findings. Sensitivity analysis was performed, by omitting one study in each turn, to investigate the influence of a single study on the overall meta-analysis estimate. Random-effects metaregression was used to examine the linear trend of the percent increase in respiratory mortality across PM_2.5_ concentrations. To maximize all the data for calculation of the pooled concentration-response, the restricted maximum likelihood (REML) approach proposed by Harbord, which provides improved estimation of the between-study variance, was used to estimate the regression coefficients [45]. Linear splines with knot at the 50th percentiles were used to assess potential nonlinear associations through metaregression analysis [46]. All analyses were performed in Stata 14.1 (StataCorp LLC, College Station, Texas, USA). A two-sided *p* value < 0.05 was considered statistically significant, except where otherwise specified.

## 3. Results

### 3.1. Search Findings and Study Characteristics

The preliminary search yielded an initial total of 1,957 publications. Following the screening of titles and abstracts based on the inclusion criteria, 22 full-text articles were included for full eligibility review and one article was identified through reference hand searching. Finally, 13 studies [18, 25, 31–41] met the search and screening criteria and were obtained for meta-analysis. Eleven studies were in English [18, 25, 31–37, 40, 41] and two were in Chinese [38, 39]. All studies are time-series studies and no population-based prospective studies or cohort studies met the inclusion criteria for further analyses in our searching period. The detailed article identification process adopting the PRISMA Flowchart model is shown in Figure 1 [30].

The identified studies were published between 2007 and 2016 and investigated major cities in China, namely, Beijing, Guangzhou, Shanghai, Shenyang, Xi'an, and Hong Kong (Table 1). The study period ranged from 1998 to 2015, of which the PM_2.5_ measurement records revealed a wide concentration range between 2 *μ*g/m^3^ and 769 *μ*g/m^3^ and the average PM_2.5_ concentration of all studied cities was far beyond the WHO AQG limits of 10 *μ*g/m^3^ for annual mean and 25 *μ*g/m^3^ for 24-hour mean of PM_2.5_ [10]. Only three studies were conducted after 2012 when China officially released PM_2.5_ data. All the studies adopted the International Classification of Diseases revision 10 (ICD-10) for the coding of the death causes of which respiratory diseases (ICD-10 codes J00–J99) including subcategories such as chronic obstructive pulmonary disease and acute respiratory infection was classified in the outcome assessment. All English articles provided risk estimates by percent increase in respiratory mortality and two Chinese publications provided RR for deaths from respiratory diseases. Unified form of risk estimates was calculated and obtained as the percent increases of respiratory mortality per 10 *μ*g/m^3^ increase in PM_2.5_ concentration. All studies provided lag-day effect adjustment for risk measurement. In particular, Li et al. [18] provided risk estimates at different lag structures of both single-day lags and multiday lags up to eight days.

### 3.2. Publication Bias and Homogeneity

**Figure 2:**
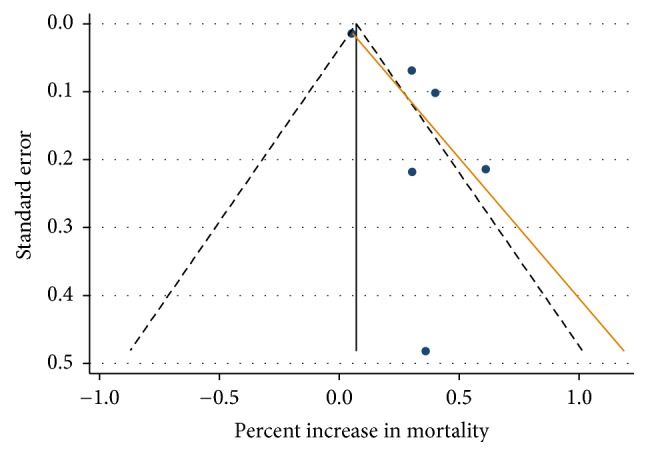
Egger's funnel plot with pseudo 95% confidence limits for single-day lags.

**Figure 3:**
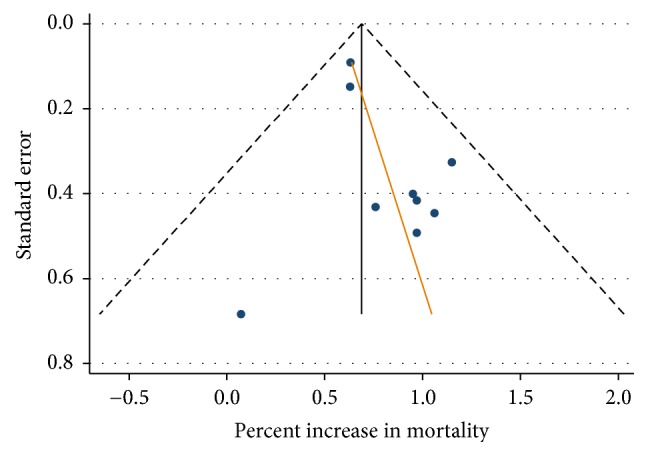
Egger's funnel plot with pseudo 95% confidence limits for multiday lags.

There was observable publication bias among the included studies for single-day lags (Egger's *p* = 0.033) but not for multiday lags (Egger's *p* = 0.120). However, the asymmetric Egger funnel plot (Figures 2 and 3) indicated potential publication bias among the studies. In view of the asymmetric funnel plots shown in Figures 2 and 3, a nonparametric “trim-and-fill” method was used to account for the publication bias in the sensitivity analysis [47, 48].

**Figure 4:**
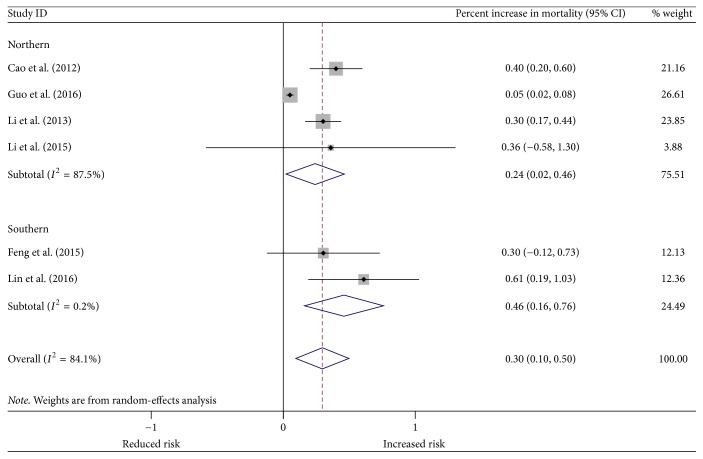
Risk estimates of respiratory mortality for single-day lags.

**Figure 5:**
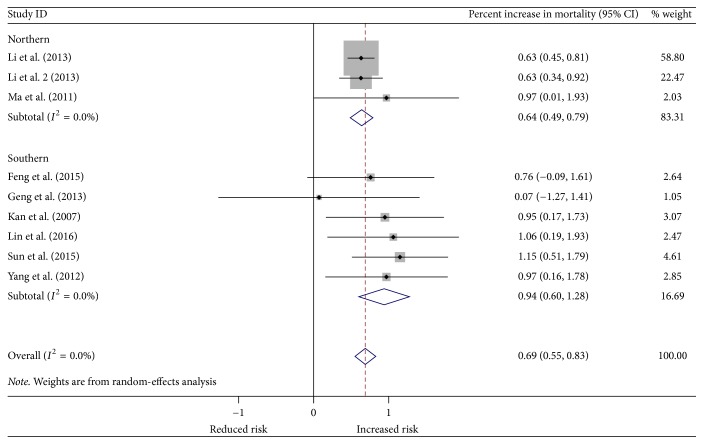
Risk estimates of respiratory mortality for multiday lags.

Statistically significant heterogeneity was found for all single-day lags by random-effects meta-analysis (*I*^2^ = 84.1%, Figure 4). Significant heterogeneity was also found for the northern cities (Figure 4). For multiday lags, no statistically significant evidence of heterogeneity was found for either all cities (*I*^2^ = 0%, Figure 5) or region-specific cities (Figure 5).

### 3.3. Association of Daily Average PM_2.5_ Concentrations with Respiratory Deaths

The combined risk estimates of included studies are shown in Figure 4 for single-day lags and in Figure 5 for multiday lags.  Table 2 summarizes the results of all the subgroup analyses. In brief, the percent increases in respiratory mortality per 10 *μ*g/m^3^ PM_2.5_ for single-day lags were 0.30 (95% CI: 0.10, 0.50), 0.24 (95% CI: 0.02, 0.46), and 0.46 (95% CI: 0.16, 0.76) for all, northern, and southern cities, respectively. There were 0.69 (95% CI: 0.55, 0.83), 0.64 (95% CI: 0.49, 0.79), and 0.94 (95% CI: 0.60, 1.28) percent increase in respiratory mortality for multiday lags in all, northern, and southern cities, respectively.

The results from subgroup analysis show that the southern cities appear having higher percent increase. However, when we examined the region effect using metaregression technique, controlling for lag structures, no statistically significant difference was found between the southern cities and the northern cities. The regression coefficient for region is −0.25 (compared to the southern cities) and corresponding 95% CI is “−0.57, 0.06.”

### 3.4. Sensitivity Analysis

For single-day lags, when omitting one study in each turn, the combined estimates of percent increase of respiratory mortality changed little (percent increase ranging from 0.25 to 0.35, Table 3). However, when trim-and-fill method was used to consider for publication bias, the overall combined effect dropped from 0.30 to 0.06.

For multiday lags, the combined estimates were more or less constant (Table 4). The percent increase ranged from 0.68 to 0.77 and the overall combined effects also changed little (from 0.69 and to 0.66). The sensitivity analysis indicated the robustness of the combined estimates for multiday lags.

### 3.5. Linear Relationship between PM_2.5_ Concentrations and Effects

**Figure 6:**
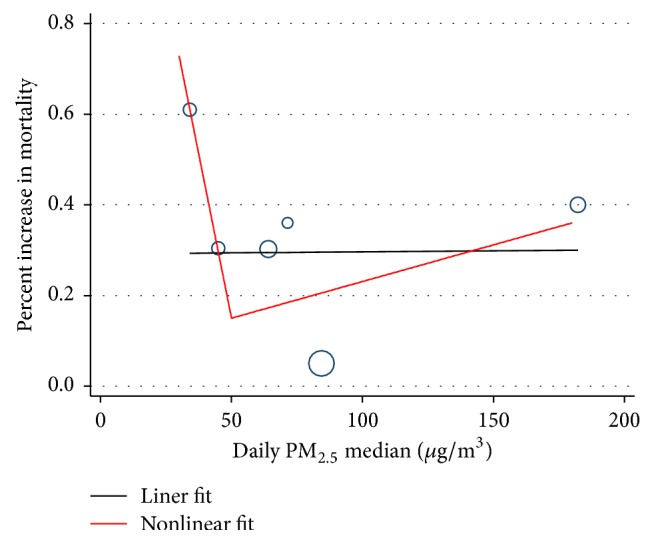
Concentration-response relationship between daily median PM_2.5_ concentration and percent increase in respiratory mortality for single-day lags.

**Figure 7:**
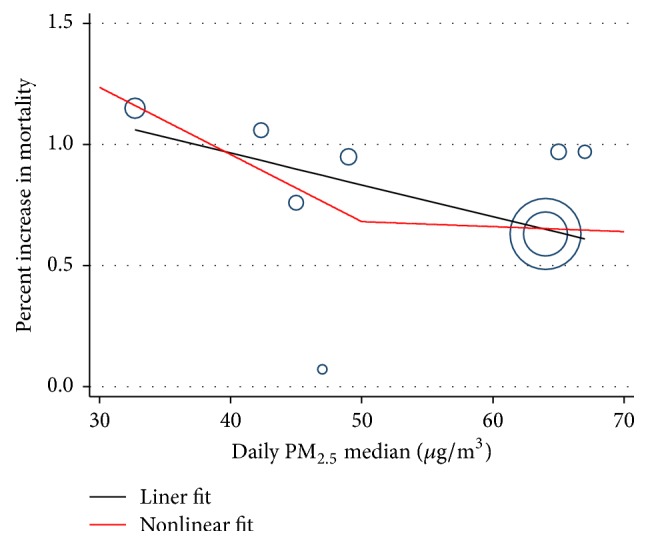
Concentration-response relationship between daily median PM_2.5_ concentration and percent increase in respiratory mortality for multiday lags.

Concentration-response relationship between PM_2.5_ concentrations and effect estimates was examined for single-day and multiday lags using metaregression model. Figures 6 and 7 showed that the percent increase in respiratory mortality kept constant with increased PM_2.5_ concentrations for single-day estimates but fell off for multiday estimates, which suggested a potential tend-to-flat pattern in the cumulative effects of PM_2.5_ on respiratory mortality at high air pollution levels observed in China. The pattern recalled the findings from previous studies that lower RR appeared with high PM_2.5_ concentration with the concentration-response curve turning flat at extremely high PM_2.5_ levels [18, 19, 22, 23]. The 95% confidence intervals of coefficients for mortality increase per 10 *μ*g/m^3^ of PM_2.5_ are “−0.055, 0.055” and “−0.333, 0.071” for single-day lags and multiday lags, respectively.

### 3.6. Nonlinear Relationship between PM_2.5_ Concentrations and Effects

Due to the limited amount of studies, nonlinear relationship between PM_2.5_ concentration and percent increase in respiratory mortality was examined using metaregression analysis with two linear splines. None of the linear splines was statistically significant (fitted splines were shown in Figures 6 and 7) and therefore no nonlinear concentration-response relationship was found across the studies.

## 4. Discussion

### 4.1. Interpretation of the Results

The results from the 13 population-based time-series studies confirmed the significant associations between PM_2.5_ concentration and respiratory mortality reported in previous studies in China. The sensitivity analysis indicated the robustness of the combined risk estimates. For single-day lags, per 10 *μ*g/m^3^ increase in PM_2.5_ concentration was associated with 0.30 (95% CI: 0.10, 0.50) percent increase in daily respiratory deaths; for multiday lags, the corresponding increase in respiratory deaths was 0.69 (95% CI: 0.55, 0.83) percent. Though not statistically significant in meta-regression analysis, difference in combined estimates was found between the northern cities and the southern cities. Combined effect estimates are relatively higher in southern cities than those in northern cities (0.46 versus 0.24 and 0.94 versus 0.64 for single-day lags and multiday lags, resp.; Table 2). Such difference suggested that PM_2.5_ might pose higher relative risk on respiratory mortality in the south region than in the north region in China. While the average daily respiratory mortality was generally lower in the south region than that in the north (Table 1), it is likely that a small increase in count of deaths would result in higher variance in mortality. The most recently published nationwide analysis in 272 Chinese cities also revealed a significant heterogeneity across different regions of China on associations between PM_2.5_ and daily mortality [28]. Limited by the small number of the studies and few cities included in our study, it is noteworthy to further investigate the possible factors behind the varying levels of PM_2.5_ effects on cause-specific mortality in different geographic regions.

No statistically significant linear or nonlinear relationship was found between the observed PM_2.5_ effects and concentrations range across the studies, which cannot provide sufficient evidence for a threshold of currently observed PM_2.5_ concentrations posing fatal respiratory effects in China. Although the results from our metaregression analysis were not statistically significant, the result is in line with previous research findings of lower RR with high PM_2.5_ concentration with the exposure-response curve turning flat at extremely high PM_2.5_ level [18, 19, 22, 23, 28].

### 4.2. Implication from This Research

To the best of our knowledge, this research is the first meta-analysis that specifically looked into the concentration-response relationship between PM_2.5_ and respiratory mortality in China. It provided an exhaustive screening of currently available literature and synthesized population-based information regarding combined risk estimate as percent increase in respiratory mortality per 10 *μ*g/m^3^ increase in PM_2.5_ concentrations. The assessment of fatal respiratory outcomes from real PM_2.5_ data in China at high concentration levels also filled in the gaps between previously projected estimates only based on extrapolated data from western countries [11, 20]. The study also responded to the evidence from a nationwide multicity investigation in China [28] as well as recommendations from similar studies conducted in India particularly on the role of fine PM and its effect on respiratory health among megacity residents [49]. By stratifying lag-day effects and regional divisions in the subgroup analysis, the research approach excluded potential confounding factors for estimates on respiratory effects. The findings from this study also recalled a positive association between PM_2.5_ exposure and lung cancer mortality as well as respiratory diseases in a Japanese cohort, where the hazard ratio for lung cancer mortality associated with a 10 *μ*g/m^3^ increase in PM_2.5_ concentrations was 1.24 (95% CI: 1.12–1.37) [21]. Though focusing on different outcomes, the Japanese study regarding PM_2.5_ and hazard ratio for mortality is a comparable reference for concentration-response associations in Asian populations.

As no specific thresholds were identified for fatal respiratory effects at observed PM_2.5_ concentrations in China from this study and with uncertainties and little evidence for damage thresholds from previous studies on a global scale [29, 50], notes should be taken that China's National Ambient Air Quality Standards [13] and WHO's AQGs values [10] may not guarantee the complete protection against adverse or fatal respiratory effects of PM_2.5_. We also hope that such findings could draw attention to public health strategies and environmental policies and call for more evidence-based decision making to address AAP at both global and local levels.

### 4.3. Strengths and Limitations

Our systematic synthesis about the associations between PM_2.5_ and respiratory mortality provides solid quantitative evidence for the evaluation and refinement of air quality guidelines and interim targets in consideration of country-specific situations and localized priorities. The study provides insights into future studies for the estimation of cause-specific mortality trends from observed and projected PM_2.5_ levels as well as implications of fatal health consequences with deteriorating AAP in LMICs. In addition to categorizing lag-day structures which were distinctive across studies, we addressed the geographical difference between the south and north regions for a more thorough interpretation of the results. Based on the findings from available literature, we noticed a lack of data availability especially in regard to geographic variations in China for the recorded study period. Nevertheless, with limited amount of available studies at current stage, we made the first step investigating the concentration-response pattern among studies with a wider variation, which shed light on future investigations of concentration-response relationship between PM_2.5_ and cause-specific mortality.

Although the meta-analysis took account of the influence of potential confounders and publication biases, the subgroup categorizations were based on prespecified confounding characteristics with limited data reported. During data extraction, we also attended to other confounders including temperature, humidity, and copollutants across studies; however, due to the limited studies, we were unable to perform further subgroup analysis. For subgroup analysis, the combined effect for single-days was not stable when considering publication bias, which warrants a cumulative meta-analysis to minimize the bias in the future. For geographic areas, in the northern cities, the data were mostly derived from the studies conducted in Beijing, while, for the southern cities, the data were mainly from Shanghai and Guangzhou. The limited cities could not let us extrapolate our findings to the larger geographic areas. We should also notice that only three included studies (Table 1) were conducted after PM_2.5_ data were systematically measured and officially published in China from 2012. Limitations on consistency of PM_2.5_ data measurement during different study period (before and after 2012) may lead to potential underestimation on risk effect associations and add more complexity for interpretation. To attend to broader data coverage as well as potential risk factors and confounders and address research gaps across existing and forthcoming literatures on PM_2.5_, future studies are needed to present a more comprehensive analysis and possibly derive a more conclusive association between PM_2.5_ and its associated health effects. We have to admit that, with the small number of analysed studies in our analysis, the possible methods for detecting publication bias are underpowered and the estimation for standard error might be poor. Therefore, the nonparametric “trim-and-fill” method developed by Duval and Tweedie was applied to account for publication bias in meta-analysis. In our study, the results for multiday lags are quite similar between trim-and-fill method and non-trim-and-fill method (Table 2). However, notable difference for single-day lags was found between the two methods (Table 2). Therefore, the bias from small number of studies cannot be ignored in our analysis. The trim-and-fill method is a rank-based data-imputation technique, which formalizes the use of funnel plots, estimates the number and outcomes of missing studies, and adjusts the meta-analysis to incorporate the imputed missing studies [51]. There exist a number of methods to estimate the number of missing studies, model the probability of publication, and provide an estimate of the underlying effect size. However, these methods are complex and highly computer-intensive to run and thus have failed to find acceptance in meta-analysis. Trim-and-fill analysis is a simple technique that seems to meet the objections to other methods and is effective and consistent with other adjusted adjustment methods [47].

In conclusion, although no statistically significant concentration-response trend was found in our meta-analysis, our study confirms and quantifies the negative association between PM_2.5_ and respiratory mortality in China. PM_2.5_ associated relative risk of respiratory mortality might be higher in certain southern cities in China. Further studies are needed to investigate the concentration-response effect of PM_2.5_ exposure on fatal health outcomes in China as well as other LMICs where AAP has been one of the major public health threats.

## Figures and Tables

**Box 1:**
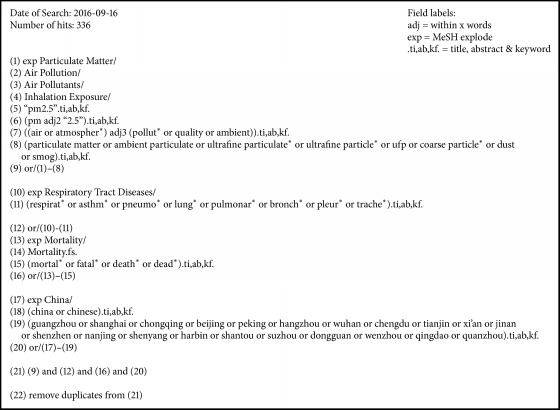
Medline (Ovid).

**Box 2:**
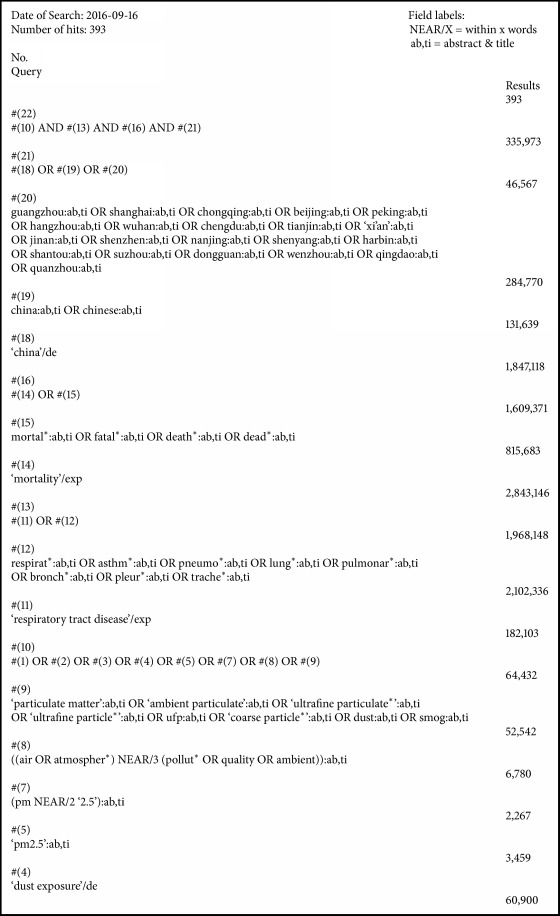
Embase (embase.com).

**Box 3:**
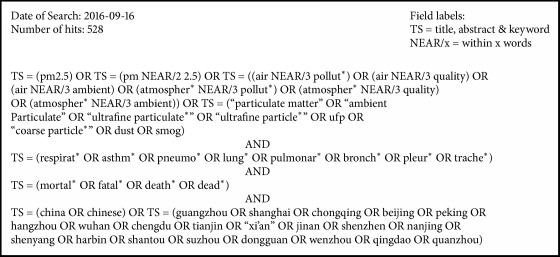
Web of Science Core Collection.

**Box 4:**
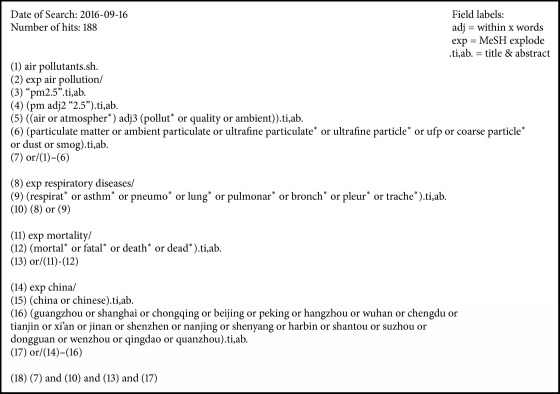
Global Health (Ovid).

**Box 5:**
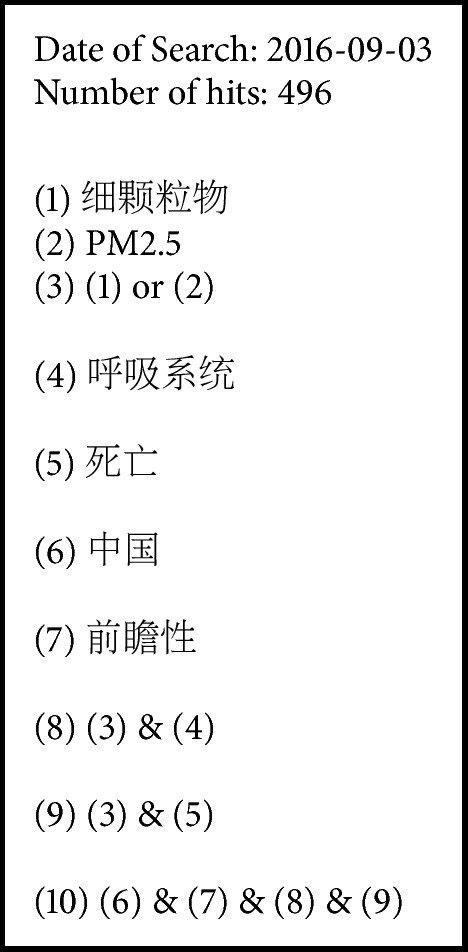
China National Knowledge Infrastructure (中国知网).

**Table 1:** Characteristics of the 13 studies included with risk estimates for PM_2.5_ concentration (*μ*g/m^3^) and respiratory mortality (RM) in China.

First author, year, city, region	Study period (duration in days)	Average PM_2.5_ concentration (min–max)	Daily RM (median)	% RM increase per 10 *μ*g/m^3^ increase in PM_2.5_	Lag-day structure
Li, 2013, Beijing, north [18]	2004–2009 (2000)	64 (2-435)	66	0.30	Single-day
Li, 2013, Beijing, north [18]	2004–2009 (2000)	64 (2-435)	66	0.63	Multiday
Li, 2015, Beijing, north [31]	2005–2009 (1826)	71.39 (20-249)	2	0.36	Single-day
Lin, 2016, Hong Kong, south [32]	1998–2011 (5113)	34 (5.8-172)	18	0.61	Single-day
Cao, 2012, Xi'an, north [25]	2004–2008 (1756)	182.2 (16.4-768.6)	7	0.4	Single-day
Lin, 2016, Guangzhou, south [33]	2012–2015 (1278)	42.3 (27.7-154)	19	1.06	Multiday
Geng, 2013, Shanghai, south [34]	2007-2008 (623)	47 (9-175)	12	0.07	Multiday
Kan, 2007, Shanghai, south [35]	2004-2005 (668)	49 (8.3-235)	12	0.95	Multiday
Ma, 2011, Shenyang, north [36]	2006–2008 (876)	67 (10-339)	6	0.97	Multiday
Yang, 2012, Guangzhou, south [37]	2007-2008 (731)	65 (12-248)	14	0.97	Multiday
Guo, 2016, Beijing, north [38]	2013 (365)	84.33 (8-471)	2	0.05	Single-day
Feng, 2015, Guangzhou, south [39]	2013-2014 (690)	45 (11.9-150)	18	0.30	Single-day
Feng, 2015, Guangzhou, south [39]	2013-2014 (690)	45 (11.9-150)	18	0.76	Multiday
Li 2, 2013, Beijing, north [40]	2005–2009 (1826)	64 (2-435)	74	0.63	Multiday
Sun, 2015, Hong Kong, south [41]	1999–2011 (4748)	32.7 (5.4-180)	18	1.15	Multiday

**Table 2:** Pooled risk estimates (percent increase in respiratory mortality [RM] per 10 g/m^3^ PM_2.5_).

Subgroup	% increase in RM (95% CI)	*I* ^2^
Single-day lags		
All	0.30 (0.10, 0.50)^a^	84.1%
All (trim-and-fill)	0.12 (−0.06, 0.31)	47.8^c^
Northern cities	0.24 (0.02, 0.46)^a^	87.5%
Southern cities	0.46 (0.16, 0.76)^b^	0.2%
Multiday lags		
All	0.69 (0.55, 0.83)^b^	0.0%
All (trim-and-fill)	0.66 (0.52, 0.79)	8.6^c^
Northern cities	0.64 (0.49, 0.79)^b^	0.0%
Southern cities	0.94 (0.60, 1.28)^b^	0.0%

^a^Random-effects model was used. ^b^Fixed-effects model was used. ^c^Cochran's  *Q*.

**Table 3:** Sensitivity analysis of single-day lags.

Study omitted	Combined estimate	95% confidence interval
Cao et al. (2012)	0.26	(0.05, 0.48)
Feng et al. (2015)	0.30	(0.08, 0.52)
Guo et al. (2016)	0.35	(0.24, 0.45)
Li et al. (2013)	0.31	(0.04, 0.57)
Li et al. (2015)	0.29	(0.09, 0.50)
Lin et al. (2016)	0.25	(0.05, 0.45)

Overall	0.30	(0.10, 0.50)

**Table 4:** Sensitivity analysis of multiday lags.

Study omitted	Combined estimate	95% confidence interval
Feng et al. (2015)	0.69	(0.55, 0.83)
Geng et al. (2013)	0.70	(0.56, 0.83)
Kan et al. (2007)	0.68	(0.54, 0.82)
Li et al. (2013)	0.77	(0.56, 0.99)
Li et al. 2 (2013)	0.71	(0.55, 0.86)
Lin et al. (2016)	0.68	(0.54, 0.82)
Ma et al. (2011)	0.68	(0.54, 0.82)
Sun et al. (2015)	0.67	(0.53, 0.81)
Yang et al. (2012)	0.68	(0.54, 0.82)

Overall	0.69	(0.55, 0.83)

## Abbreviations

AAP: Ambient Air Pollution
AQGs: Air Quality Guidelines
CI: Confidence interval
CO: Carbon monoxide
DALYs: Disability-adjusted life-years
GBD: Global Burden of Disease
ICD-10: International Classification of Diseases revision 10
LMICs: Low- and Middle-Income Countries
MeSH: Medical subject heading
MOOSE: Meta-Analysis of Observational Studies in Epidemiology
NO_2_: Nitrogen dioxide
O_3_: Ozone
PM: Particulate matter
PM_2.5_: Particulate matter with aerodynamic diameter of 2.5 micrometres or less
PM_10_: Particulate matter with aerodynamic diameter of 10 micrometres or less
PRISMA: Preferred Reporting Items for Systematic Reviews and Meta-Analysis
RR: Relative risk
SE: Standard error
SO_2_: Sulfur dioxide
WHO: World Health Organization.
